# Reappraising the utility of Google Flu Trends

**DOI:** 10.1371/journal.pcbi.1007258

**Published:** 2019-08-02

**Authors:** Sasikiran Kandula, Jeffrey Shaman

**Affiliations:** Department of Environmental Health Sciences, Columbia University, New York, New York, United States of America; University of Trento, ITALY

## Abstract

Estimation of influenza-like illness (ILI) using search trends activity was intended to supplement traditional surveillance systems, and was a motivation behind the development of Google Flu Trends (GFT). However, several studies have previously reported large errors in GFT estimates of ILI in the US. Following recent release of time-stamped surveillance data, which better reflects real-time operational scenarios, we reanalyzed GFT errors. Using three data sources—GFT: an archive of weekly ILI estimates from Google Flu Trends; ILIf: fully-observed ILI rates from ILINet; and, ILIp: ILI rates available in real-time based on partial reporting—five influenza seasons were analyzed and mean square errors (MSE) of GFT and ILIp as estimates of ILIf were computed. To correct GFT errors, a random forest regression model was built with ILI and GFT rates from the previous three weeks as predictors. An overall reduction in error of 44% was observed and the errors of the corrected GFT are lower than those of ILIp. An 80% reduction in error during 2012/13, when GFT had large errors, shows that extreme failures of GFT could have been avoided. Using autoregressive integrated moving average (ARIMA) models, one- to four-week ahead forecasts were generated with two separate data streams: ILIp alone, and with both ILIp and corrected GFT. At all forecast targets and seasons, and for all but two regions, inclusion of GFT lowered MSE. Results from two alternative error measures, mean absolute error and mean absolute proportional error, were largely consistent with results from MSE. Taken together these findings provide an error profile of GFT in the US, establish strong evidence for the adoption of search trends based 'nowcasts' in influenza forecast systems, and encourage reevaluation of the utility of this data source in diverse domains.

## Author summary

Google Flu Trends (GFT) was proposed as a method to estimate influenza-like illness (ILI) in the general population and to be used in conjunction with traditional surveillance systems. Several previous studies have documented that GFT estimates were often overestimates of ILI. In this study, using a recently released archive of data of provisional incidence from a large surveillance system in the US (ILINet), we report errors in GFT alongside errors from ILINet’s initial estimates of ILI. This comparison using information available in real-time allows for a more nuanced assessment of GFT errors. Additionally, we describe a method to correct errors in GFT and show that the corrected GFT estimates are at least as accurate as initial estimates from ILINet. Finally, we show that inclusion of corrected GFT while forecasting ILI in the next four weeks considerably improves forecast accuracy. Taken together, our results indicate that the GFT model could have added value to traditional surveillance and forecasting systems, and a reevaluation of the utility of the underlying search trends data, which is now more openly accessible, in fields beyond influenza is warranted.

This is a *PLOS Computational Biology* Methods paper.

## Introduction

Surveillance of seasonal influenza and other respiratory illnesses deservedly receives significant attention from public health agencies in the United States. To complement traditional surveillance systems, both internet- [1–7] and non-internet-based [8–11] proxy indicators of incidence have been developed. Among these, of note is Google Flu Trends (GFT) [1, 12], which estimated influenza-like illness (ILI) from online search activity. GFT estimates from an initial model and subsequent revisions to the model were publicly available until 2015, when the service was discontinued [13]. Although Google has not offered reasons for the termination, one contributing factor could well have been the widely reported propensity of GFT to over-estimate ILI, which effectively morphed it in the public perception from a poster child for the power and utility of big data to one of its hubris [14–20].

However, this perception is probably misplaced. The most comprehensive and commonly cited study of GFT errors for locations in the United States was published by Lazer et al [14], following an anomalous season during which the errors were much larger than previously observed. These findings were supported by several other studies that were smaller in scope but reported errors of approximately the same magnitude at different locations and geographical resolutions [21, 22]. In this paper, using newly available surveillance data, we revisit GFT estimates for locations in the US and show that its errors are less substantial than previously reported.

The severity of a respiratory viral infection in an individual depends on multiple factors, and in most cases the symptoms are mild and do not require medical attention. As a consequence, the more widely used surveillance systems in the US–the Centers for Disease Control and Prevention (CDC)’s ILINet and FluSurv-NET systems, for example–only capture infections that are severe enough to precipitate a visit to a physician's office or hospital. On the other hand, the relationship between the severity of a respiratory infection and the likelihood that an individual initiates an online search session for related information, is unknown; hence, the signals that drive GFT and the surveillance systems are intrinsically different. Nonetheless, as GFT used incidence data from ILINet as its response variable, it has been a common practice, and one that we follow in this study, to use these rates as reference or ground truth when reporting the accuracy of GFT estimates.

However, in reporting US national and regional errors, most previous studies, including Lazer et al [14], did not account for delayed reporting to ILINet. The fully observed ILINet rates (ILIf) are finalized no sooner than 2–3 weeks after the conclusion of a surveillance week, as some of the surveillance network data are submitted late, and in some instances, revisions can even occur several months later. The rates released in the interim are estimates based on partial observations (ILIp) and the magnitude of difference between ILIp and ILIf, as we report here, varies by location, influenza season and the phase of a season.

Given these reporting delays and revisions, it is ILIp rather than ILIf that informs real-time decisions. Thus, a more appropriate error analysis, one that better reflects an operational scenario, should compare errors of GFT (ILIf—GFT) to errors of ILIp (ILIf—ILIp). An archive of ILIp at US national and Health and Human Service (HHS)[23] regional levels for 6 seasons has been made available [24] recently, and in this study we used this archive to recompute GFT and ILIp errors[25, 26]. Additionally, we extended the analysis to finer geographical resolutions as ILIf is now also available for US states. Finally, we report for the first time, errors from the final GFT model, updated in fall 2014, before the service was discontinued.

Google's recent initiative to provide access to its search trends through an API[13] supports more open data sharing. This effectively decouples data from model and facilitates the development of alternative models to GFT. Through the analysis described here, we hope to establish an error profile of GFT that can serve as a baseline for comparing these alternative models.

More importantly, although GFT was proposed by its developers as a supplement to traditional surveillance systems and not a replacement, the focus to date has been disproportionately on evaluating GFT's ability to mimic surveillance systems rather than on evaluating its utility when deployed in conjunction with these systems in operational settings. Previous findings suggest that the errors in GFT can be reduced by combining GFT estimates with lagged surveillance rates [14, 27, 28]. Here we propose a similar remedial step with a parsimonious regression model and show that the corrected GFT is more accurate than ILIp.

A natural extension is to assess whether GFT, its errors thus corrected, could have improved longer term forecasts by providing more timely outbreak information than traditional surveillance systems. For this purpose, we generated forecasts of ILI one to four weeks in the future using ILIp alone, and using both ILIp and error corrected GFT. We demonstrate that the inclusion of GFT considerably improves the accuracy of near-term forecasts and thus adds value to traditional surveillance systems.

## Materials and methods

In this section we describe in detail the two data sources used—an outpatient surveillance system and GFT—access information for the two sources, and the measures used to calculate errors of these estimates. We then describe the autoregressive model framework used to generate near term forecasts, followed by details of the forecast generation and validation process.

### US influenza outpatient surveillance network (ILINet)

The ILINet surveillance system [29], developed and supported by the CDC, collects data from nearly 3000 healthcare providers in the US on outpatient visits for ILI, which is defined as fever (temperature above 100*°*F) co-occurring with cough and/or sore throat. Weekly counts of patients seen for ILI and for any reason are submitted to the system. These count data are used to calculate the percentage of outpatient visits due to ILI. In this study, by ILI rate we refer to population-weighted aggregates of ILI.

A Morbidity and Mortality Weekly Report (MMWR)[30] surveillance week runs from Sunday thru Saturday and aggregated ILI rates at US state-, HHS regional- and national levels are publicly released through the *FluView* [31] website on Friday (6 days after a week concludes). The system allows for delayed reporting from providers and the delayed data are included in subsequent weekly releases. Hence, the ILI estimates for a week can change for multiple weeks following initial release. We refer to the ILI rates calculated from incomplete reporting as partially observed ILI rates, and in this paper denote the rates as per the first week of release as ILIp. An archive of revisions for the 2009/10 season onwards has been recently made available [24, 32] and for the 2013/14 season and later, these data are also accessible through the DELPHI group's *epidata* API [33].

Although ILIf is available for the US, HHS regions and states, ILIp is not currently available at the state level. ILI rates for the 2009/10 to 2014/15 seasons that were available on *FluView* at the end of surveillance week 20 of the 2017/18 season (May 13–19, 2018) were considered to be ILIf. This date is over two years after the end of the time period studied, and hence we assume that it is very unlikely that these rates would be further revised. Note that both ILIp and ILIf are rates, and ILIp can over or underestimate ILIf.

### Google Flu Trends (GFT)

Originally developed in 2008, GFT estimated ILI rates in a population based on the frequency of a selected set of queries to the Google search engine [1]. The 2008 model used 45 queries, whose search frequencies were historically well correlated [34, 35] with ILI rates, as explanatory variables. To generate the estimates for the US, ILI rates were used as the response variable in the model. In response to observed deficiencies in the predictions, revisions to the model, including updates to the feature set, were made in 2009, August 2013 and August 2014. GFT estimates that were published in real-time from September 2008 through August 2015, along with estimates from revised models applied to past seasons continue to be hosted publicly [12].

**Fig 1:**
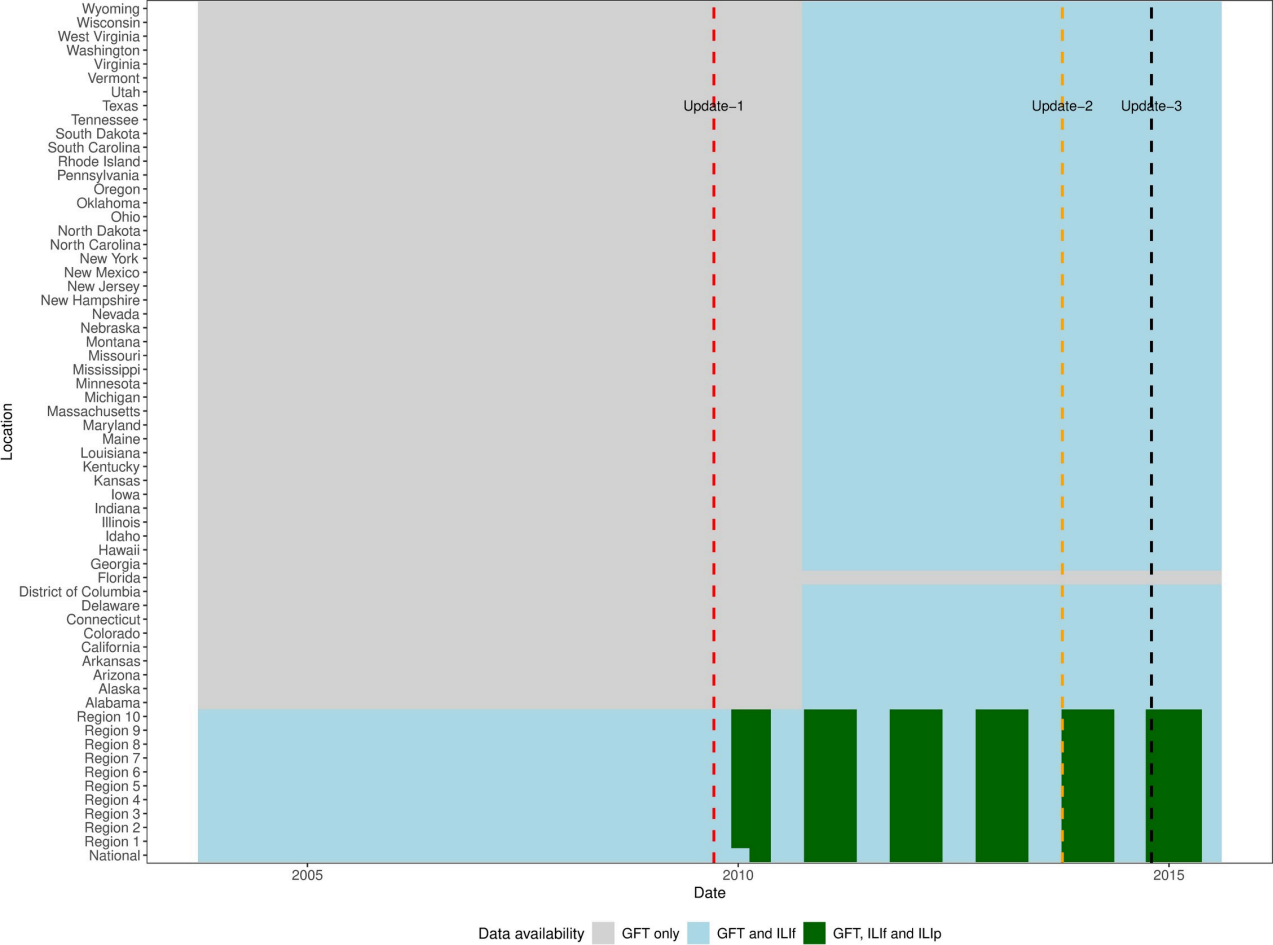
Availability of GFT, ILIf, and ILIp at US national, regional and state levels in the US. At the regional level, GFT and ILIf were available from 2003, and ILIp were available from 2009/10 season onwards, excluding off-season weeks. For states, ILIp were never available to the public, and ILIf is available from 2011/12 season onwards. Updates to GFT model are indicated by the vertical lines.

Fig 1 shows the availability of GFT, ILIf and ILIp at different locations in the US. For US and HHS regions, GFT, ILIf and ILIp are available for six seasons—2009/10 to 2014/15—and for the states ILIf and GFT are available for the last 5 of these 6 seasons. The vertical lines indicate the time points of revisions to the GFT model; therefore estimates for seasons 2009/10 thru 2012/13 seasons, season 2013/14, and season 2014/15 are from different models.

Unlike ILINet, GFT estimates for a week are finalized at the end of the week. Furthermore, as the GFT estimates were completely automated, and computed in real-time, they did not have the 6-day lag between the end of a week and the release of data as is the case with ILINet. This translates to GFT providing weekly incidence estimates for at least one more week than ILINet, at any given point of time. The estimate for this one additional week is sometimes referred to as a nowcast.

### Error measures

For each week and location, error is defined as $y-\hat{y}$, where *y* is the reference, ILIf, and $\hat{y}$ the estimate from GFT or ILIp. Aggregate error measures, Mean Squared Error (MSE), Mean Absolute Error (MAE) and Mean Absolute Proportional Error (MAPE) are respectively the mean of the square of errors, of the absolute error and of absolute error as a proportion of the reference value, and are reported across all seasons and locations, or for each season (across all location) and each location (across all seasons). During the study period, the reference value was never zero, and hence MAPE was computable. Formally,

$$MSE=\frac{1}{n}\sum_{i=1}^{n}{\left(y_{i}-{\hat{y}}_{i}\right)}^{2}$$

$$MAE=\frac{1}{n}\sum_{i=1}^{n}\left|y_{i}-{\hat{y}}_{i}\right|$$

$$MAPE=\frac{1}{n}\sum_{i=1}^{n}\frac{\left|y_{i}-{\hat{y}}_{i}\right|}{y_{i}}$$

As the errors in 2012/13 are reportedly much larger than during the other seasons included in the study, inclusion of this season could obscure overall results, and hence we report aggregate measures both with and without this season.

### Seasonal autoregressive integrated moving average (ARIMA) model

A non-seasonal ARIMA model is specified by three parameters—*p*, the order of the autoregressive component; *q*, the order of the moving average component; and *d*, the degree of differencing required to make the given time series stationary. For a time series, *Y*, let *y* denote the time series obtained by *d* degree differencing. Thus, an *ARIMA(p*, *d*, *q)* is a model of the form:

$$y_{t}=c+\phi_{1}y_{t-1}+\cdots +\phi_{p}y_{t-p}+\theta_{1}\varepsilon_{t-1}+\cdots +\theta_{q}\varepsilon_{t-q}$$

where the elements, ε_i_, represent the forecast errors at the *i*^*th*^ time step. Elements *c*, *ϕ*_*1*_, *…*, *ϕ*_*p*_, *θ*_*1*_, …,*θ*_*q*_ can be estimated through maximum likelihood estimation. As influenza in the US has strong yearly seasonality, a seasonal ARIMA model may often, though not always, be a better fit. Seasonal ARIMA models are specified with three additional parameters *P*, *D*, *Q* where *D* denotes seasonal differencing and *P*, *Q* are analogous to *p*, *q*, respectively, as defined above.

We used an implementation of an iterative method proposed by Hyndman and Khandakar [36] from the R [37] *forecast [38]* package to find an appropriate order for the ARIMA models. Briefly, the Kwiatkowski-Phillips-Schmidt-Shin (KPSS) test [39] and extended Canova-Hansen test [40] are used to determine an appropriate *d* and *D* respectively. To find values for the remaining parameters, an iterative process is initiated with the model that has the lowest Akaike’s Information Criterion (AIC) [41] amongst a small default set of models, as the candidate model. In each subsequent step, the parameters of the candidate model are varied by ±1 within a pre-specified parameter space (*p*, *q*: (0, 5); *P*, *Q*: (0, 2)) and the variant with the lowest AIC becomes the new candidate model. The process is terminated when the parameter space is exhausted or all variants of the candidate model result in a higher AIC.

### Error correction and forecast generation

Retrospective near-term forecasts were generated for US National and the 10 HHS regions during the 2010/11 to 2014/15 influenza seasons for MMWR weeks 41 through week 20. Traditionally an influenza season is considered to run from MMWR week 40 thru MMWR week 39 of the following calendar year. Late spring and summer weeks (MWWR week 20 onwards) experience low incidence and hence were excluded in this study. Separate models were fit for each location and week. Models for each location are isolated as they do not use observations from any other location.

Let *X*_*i*_ and *Z*_*i*_ denote the log transformed ILI rates and GFT estimates at week *i* respectively. All ILI/GFT values less than 2 (per 100000) were rounded up to 2 before log transformation. As described in a previous section, when forecasts are generated operationally at the end of week *t*, *X*_1_,⋯,*X*_*t*_ and *Z*_1_,⋯,*Z*_***t*+1**_ would be available; *i = 1* indicates MMWR week 40 of 2009/10 season. For a given week *w ≤ t*, *X*_*w*_ is ILIp if *w* and *t* belong to the same season, and ILIf otherwise. Corrected GFT, ${\hat{Z}}_{t+1}$, is estimated using a random forest [41–44] regression model with explanatory variables *X*_*t*_,*X*_*t*−1_,*X*_*t*−2_,*Z*_*t*+1_,*Z*_*t*_,*Z*_*t*−1_,*Z*_*t*−2_ and response variable *X*_*t*+1_. S4 Fig shows corrected GFT at the US national level, and its error with respect to ILIf.

To generate near term forecasts, two models were developed: the first using ILIp only (*ILIp*), and the second using ILIp and corrected GFT (*ILIp+GFT*). For a given week *t*, the *ILIp* models were trained on the time series *X*_*1*_, *…*, *X*_*t*_ and used to forecast rates for weeks *t+1*, *…*, *t+4*, denoted by ${\hat{X}}_{t+1}\ldots {\hat{X}}_{t+4}$. The corresponding *ILIp+GFT* ARIMA models were fit using the time series $X_{1},\ldots ,X_{t},{\hat{Z}}_{t+1}$ and forecast rates for ${\hat{X}}_{t+2}\;\ldots \;{\hat{X}}_{t+4}.\;{\hat{Z}}_{t+1}$ doubles as the 1-week ahead forecast. For both models MSE, MAE and MAPE, as defined above, were calculated with ILIf as reference.

For example, the *ILIp* models for week 46 of 2011/12 season were fit using ILIf from the 2009/10 and 2010/11 seasons and ILIp from weeks 40 to 46 of the 2011/12 season, and were used to forecast rates for weeks 47 through 50. The GFT correction model for week 46 was fit using training instances compiled with ILI and GFT through week 46 of 2011/12 season and used to estimate, ${\hat{Z}}_{47}$ with test instance (*X*_46_,*X*_45_,*X*_44_,*Z*_47_,*Z*_46_,*Z*_45_,*Z*_44_). *ILIp+GFT* models used ${\hat{Z}}_{47}$ as an additional observation, and forecast rates for weeks 48 to 50. Therefore, the week 50 forecast from *ILIp* ARIMA model was a 4-week ahead forecast but a 3-week ahead forecast for the *ILIp+GFT ARIMA* model. Forecast errors for both model forms were then calculated using ILIf for weeks 47 to 50 as reference.

## Results

### GFT as an estimator of ILIf

Table 1 shows that the MSE of GFT is on average 2.5 times that of ILIp with considerable variability by location. Region 9, where the mean squared errors were nearly equal, had the smallest difference between GFT and ILIp, whereas Region 4 had the largest difference, with GFT error about 7.6 times as large as that of ILIp. Similar variability was observed across seasons, with the largest difference by far occurring during the 2012/13 season, and the smallest during 2009/10. As previously reported [14], GFT estimates for weeks around the peak of the 2012/13 season were large over-estimates, which contributed considerably to the high mean errors.

**Table 1:** Aggregated squared error. Mean (standard deviation, [25th–75th percentile]) for the entire study period, disaggregated by location and season. US national has ILIp has 11 fewer dates than the regions. Overall and location aggregations exclude 2012/13 season.

	GFT	ILIp
Overall	0.364 (0.94, [.02–.35])	0.143(0.4, [0–0.09])
National	0.138 (0.24, [0–.17])	0.031 (0.05, [0–.04])
Region 1	0.101 (0.16, [.01–.14])	0.04 (0.1, [0–.04])
Region 2	0.515 (0.76, [.06–.67])	0.185 (0.39, [.01–.14])
Region 3	0.342 (0.4, [.07–.47])	0.066 (0.11, [.01–.08])
Region 4	0.282 (0.58, [.02–.21])	0.037 (0.1, [0–.03])
Region 5	0.147 (0.22, [.01–.19])	0.024 (0.04, [0–.03])
Region 6	0.714 (2.18, [.03–.68])	0.181 (0.38, [.01–.17])
Region 7	0.601 (1.43, [.02–.4])	0.112 (0.29, [.01–.09])
Region 8	0.137 (0.31, [.01–.15])	0.042 (0.07, [0–.07])
Region 9	0.695 (0.88, [.07–.9])	0.681 (0.89, [.05–.92])
Region 10	0.337 (0.53, [.05–.39])	0.17 (0.37, [0–.15])
2009/10	0.274 (0.47, [.03–.26])	0.191 (0.45, [.01–.15])
2010/11	0.545 (0.97,[.03–.56])	0.181 (0.44, [0–.12])
2011/12	0.353 (0.45, [.06–.45])	0.158 (0.5, [0–0.07])
2012/13	5.847 (15.77, [.11–2.66])	0.175 (0.6, [0–0.08])
2013/14	0.29 (0.59,[.01–.33])	0.083 (0.21, [0–0.05])
2014/15	0.338 (1.54,[.01–.18])	0.119 (0.33, [0–0.08])

The corresponding difference in MAPE (S1 Table) is slightly smaller overall (GFT error 1.8 times ILIp error), with the GFT error actually lower than that of ILIp for Region 9. In reporting Table 1 (and S1 Table) we excluded season 2012/13 for *Overall* and regional aggregations; see S2 Table for aggregations across all seasons.

**Fig 2:**
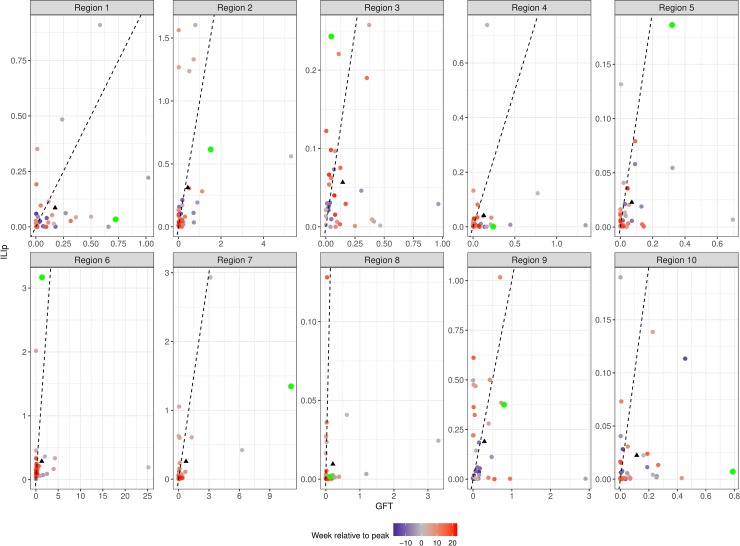
Squared errors from GFT and ILIp for HHS regions during the 2014/15 season. The green data points show the error during the week of maximum weekly ILIf—the peak week—and the remaining data points are color coded by their distance from peak week. The black triangles show the mean error for the season. The black line is the *y = x* line; points below this line have larger errors from GFT than from ILIp. In all regions, the mean error from GFT falls below the line.

Fig 2 shows MSE with the final version of the GFT system for the 2014/15 season and the average GFT errors in all regions (denoted by the black triangle) are larger than corresponding ILIp errors. But as indicated by the data points above the diagonal, ILIp does not consistently have lower errors for all weeks. As supported by S2 Fig, during weeks very early (blue data points) or towards the end (red data points) of the season, the difference between GFT and ILIp is relatively small (data points closer to the diagonal). The larger errors for both ILIp and GFT occur during weeks of increased ILI activity around the peak week (green and grey data points). S1 Fig has the corresponding MAPE errors for the 2014/15 season. On the whole, errors during the 2014/15 season are in line with some of the previous seasons, and the final GFT model was not a marked improvement over previous models.

**Fig 3:**
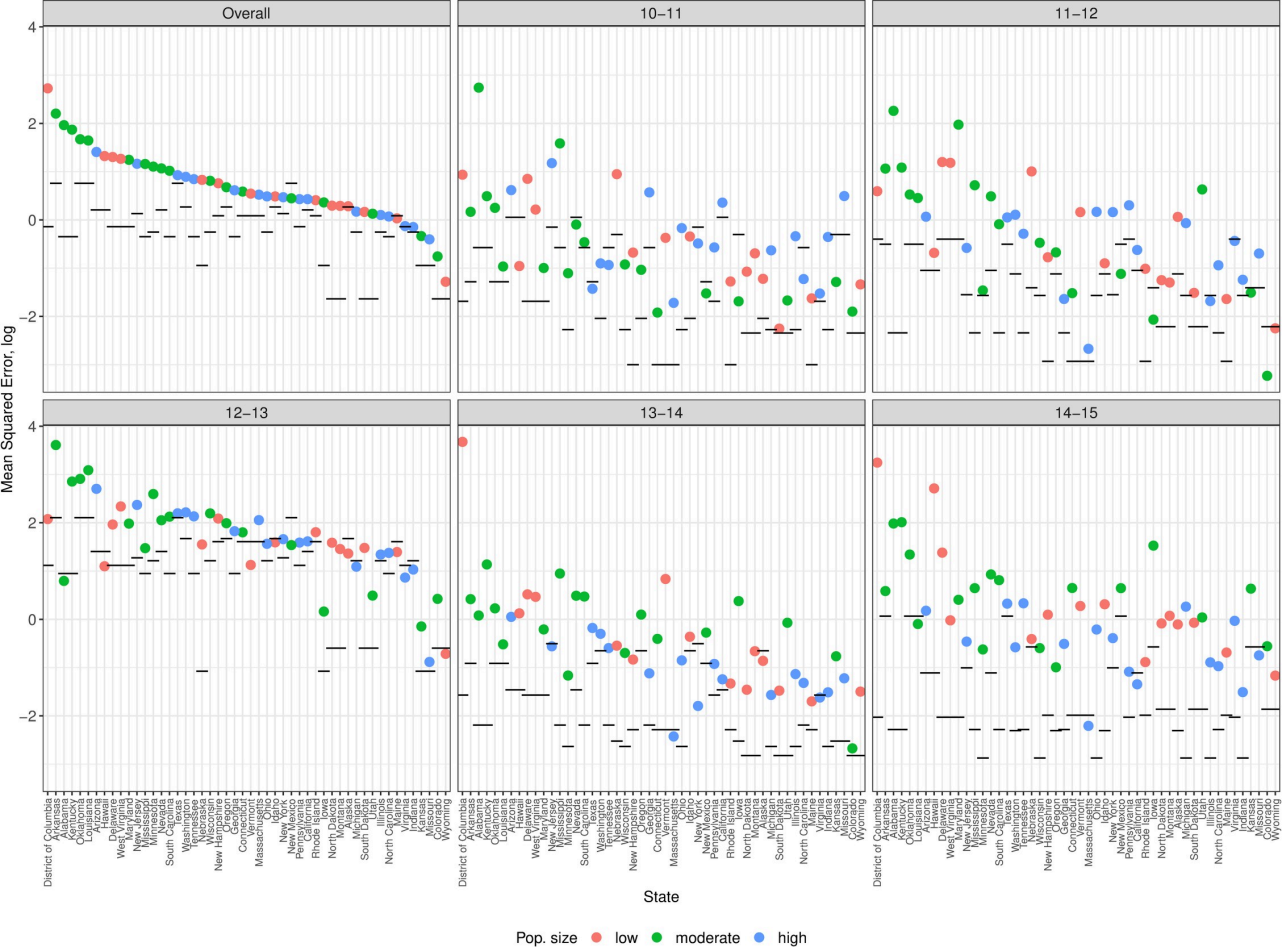
Mean squared error of GFT observed in US states. The top left panel, *Overall*, shows average errors across 5 seasons and each of the other panels is limited to one season. The data points are color coded by population size and ordered by *overall* error (high to low). The black line shows the errors from corresponding HHS regions.

Looking at GFT errors at the state-level (Fig 3, S3 Fig), the errors are much larger than the errors at the corresponding HHS regions (black horizontal mark). Overall (top left panel), states with low (< 2 million) and medium (2–6 million) population sizes, tend to have larger GFT errors than high population states. We know from previous work [5] with search trends from Google's Health Trends API, that terms/queries whose search frequencies do not meet a predetermined threshold limit are reported as 0. If GFT used a dataset that was based on similar criteria, low population states where search volumes are smaller, would have had sparser feature spaces.

Similar patterns were seen when the errors are disaggregated by season. It is interesting to note that among all seasons studied, the season with the smallest differential in MSE between state and regional errors was the anomalous 2012/13 season, where the large increase in GFT regional errors was not accompanied by a proportionate increase in errors for states. In a few cases, the errors for a state were smaller than the errors at the corresponding region.

### Nowcast using lagged ILIp and GFT

As shown in Table 2 and S4 Fig, considerable reduction in GFT MSE was achieved through regression on lagged data. An overall reduction of 44% was observed across the 11 locations and 4 seasons. The large reduction during 2012/13 reiterates the utility of this additional step as a check against extreme failures of GFT. It is also interesting to note that this step reduces GFT errors below that of ILIp i.e. the use of search trend data can not only provide an estimate of incidence a week earlier than ILINet, but can do so more accurately than ILINet's own initial estimate of incidence. S3 Table shows the corresponding overall reductions in MAE and MAPE, and the findings noted with MSE hold.

**Table 2:** Mean squared error in one-week ahead estimates. *Change* column indicates percentage reduction in mean error by regressing ILIf on lagged ILIp+GFT. 2012/13 was excluded while aggregating overall and by region. Paired Wilcoxon signed rank tests for the hypothesis that the median of errors in GFT (Z) are *greater* than the median of errors in corrected GFT ($\hat{Z}$) were performed; cases where p > .05 are denoted by an asterisk (*).

	ILIp	GFT (Z)	Corrected GFT ($\hat{Z}$)	Change,% $(Z-\hat{Z})/Z$
Overall	.301	.382	.215	44
National	.121	.148	.085	43
Region 1	.068	.116	.061	47
Region 2	.294	.603	.246	59
Region 3	.264	.378	.224	41
Region 4	.254	.306	.174	43
Region 5	.180	.147	.122	17
Region 6	.678	.804	.417	48
Region 7*	.334	.600	.372	38
Region 8	.147	.151	.094	38
Region 9	.714	.601	.307	49
Region 10	.258	.354	.259	27
2010/11	.317	.546	.214	61
2011/12	.187	.355	.152	57
2012/13	.431	5.753	.617	89
2013/14	.273	.290	.221	24
2014/15*	.420	.342	.268	22

There is considerable variability in the magnitude of improvement in nowcast quality across locations and seasons, and with a few exceptions the decrease in errors was significant (P < 0.05) per a paired Wilcoxon signed rank test [45–47].

### Near-term forecasts using nowcasts

**Fig 4:**
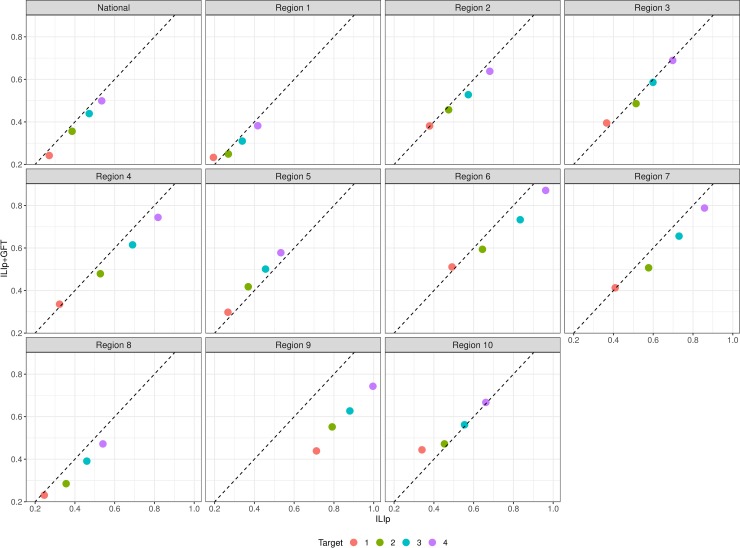
Mean squared error of near term forecasts for *ILIp* and *ILIp+GFT* models. The data points are color coded by target. Points below the diagonal (broken black line) indicate instances where forecast quality improved with the use of GFT. Each panel is for one of the locations.

Table 3 shows the MSE for near-term forecasts generated with ILIp alone and using both ILIp and corrected GFT (*ILIp+GFT*). At all targets (1- to 4-week ahead estimates) and seasons, and for all but two regions (Fig 4), inclusion of GFT lowered MSE. The overall MAPE with the *ILIp+GFT* models is also lower (S4 Table, S5 Fig), although the relative advantage over *ILIp* with different regional or seasonal disaggregation criteria is more mixed. The overall reduction in errors when aggregated by target or region is not limited to reduction from the anomalous 2012/13 season; *ILIp+GFT* errors continue to be lower and significant when the 2012/13 season is excluded (S5 Table).

**Table 3:** Mean squared error of near-term forecasts. *ILIp* was generated with ILIp alone and *ILIp+GFT* by appending corrected GFT to ILIp. The lower error in each row is underscored. P-values from a paired Wilcoxon signed rank test that the median of error in ILIp forecasts are *greater* than the median of errors in *ILIp+GFT* forecasts are also shown; cases where p > .05 are denoted by an asterisk (*).

	ILIp	ILIp + GFT	p
Overall	0.761	0.605	< .001
1 week ahead	0.327	0.294	.01
2 week ahead	0.611	0.459	< .001
3 week ahead	0.907	0.700	< .001
4 week ahead	1.199	0.968	< .001
National	0.452	0.367	< .001
Region 1	0.248	0.221	< .001
Region 2	0.605	0.503	.01
Region 3*	0.857	0.764	.25
Region 4	0.969	0.685	.03
Region 5*	0.526	0.582	.67
Region 6	1.554	1.233	< .001
Region 7	1.013	0.810	< .001
Region 8	0.445	0.316	< .001
Region 9	1.149	0.608	< .001
Region 10*	0.553	0.568	.76
2010/11	0.747	0.549	< .001
2011/12	0.268	0.229	< .001
2012/13*	1.035	0.866	.24
2013/14	0.643	0.549	< .001
2014/15	1.091	0.819	< .001

For all three measures, the accuracy of the regression model's nowcast either matches or exceeds that of the 1-week ahead ARIMA forecast. Reduction of errors at longer horizons is larger and this is quite likely due to the *k* week ahead forecast of the *ILIp+GFT* model being lined up with the *k+1* week ahead forecast of the *ILIp* model, as ARIMA errors tend to increase with increasing horizon.

## Discussion

The increasing availability of big data has naturally led to the development of experimental applications in several domains, including those such as public health surveillance that have traditionally relied on more robust, but also labor intensive, data collection processes. Google Flu Trends was developed as an alternative method to measure ILI in the general population, to be used in conjunction with traditional surveillance methods when and where they exist. Given its prospects for use (and misuse) GFT appropriately received wide attention; but it is our belief that it has been adjudged wanting against goals it was not designed to meet.

Reporting errors of ILIp rates alongside GFT errors, helps quantify the transient errors in ILINet due to delayed reporting and provides a more appropriate baseline for comparing the accuracy of GFT (and alternative nowcast models) in operational settings. The use of ILINet rates as ground truth, here and in previous studies, is appropriate simply because these are the targets GFT was designed to estimate and a more reliable system for estimating ILI broadly in the US does not exist. However, when assessing the validity of alternatives methods for influenza estimation, we must remain cognizant of the deficiencies of ILINet in capturing influenza transmission at metapopulation scales–for instance, its passive data collection process, broad symptom definition that is geared towards ILI rather than influenza, and estimation of incidence from visit counts without a requirement for virologic confirmation.

The opening up of Google Trends API directly addresses one major obstacle in improving nowcasts over the GFT models, namely, the non-availability of public search trends data. Additionally, US state level ILINet rates were not available prior to the 2017/18 season, and previously required some form of extrapolation from regional ILI rates to state ILI rates in order to build state-level nowcast models. With these data now being released in real time, nowcast models for states should be able to identify more reliable predictor variables, and the accuracy of these nowcast estimates can be expected to improve over GFT estimates. Furthermore, fine-grained nowcast estimates, say at city or county scales, or for large hospital settings, are possible when reliable ILI rates exist.

Our results show that a regression model with lagged ILIp and GFT predictors can adequately correct errors in search trend based nowcasts and thereby avoid catastrophic failures, and the model estimated rates are at least as accurate as partially observed surveillance rates in the US. Indeed, during the 2017/18 and 2018/19 influenza seasons, which saw atypical, large, sustained outbreaks, our search trends based nowcasts did not exhibit large errors. Use of this data source alongside other data sources like twitter, electronic medical records, Wikipedia logs etc. [3, 4], can further reduce the risk of such failures by making the nowcasts less reliant on any single source.

Results with the near-term forecasts show that the provision of an additional week of observation to the ARIMA models considerably improves forecast quality. Forecasts generated with ILIp and corrected GFT also improve over those generated with ILIp and uncorrected GFT (S6 Table). Both the random forest and ARIMA models used here were standard implementations from open source statistical packages with no domain specific tailoring, and we have no reason to believe that these improvements and the ensuant findings are specific to the models used. Other mechanistic or time series models may offer similar improvements in accuracy, and some recent results are suggestive of such improvements [48, 49]. Our choice of ARIMA as the forecast model should not be construed as a vote in favor of its optimality in forecasting ILI; on the contrary, as ARIMA is not informed by any of the transmission dynamics of ILI, we include it as a non-naïve reference method. Researchers proposing alternative methods tailored for ILI should be expected to show that they do at least as well as ARIMA.

Overall, we believe that the results presented here provide sufficient evidence to encourage continued efforts to improve search trend based nowcasts for influenza and make a case for their more wide-spread adoption in operational forecasting systems. At a minimum, they show that reports of the failure of GFT are not unequivocal and they should not deter use of Google Trends API in areas other than ILI estimation.

## Supporting information

S1 Table Aggregated absolute error and absolute Proportional Error. Mean (standard deviation, [25th–75th percentile]) for the entire study period, disaggregated by location and season. US national has ILIp has 11 fewer dates than the regions. Overall and location aggregations exclude 2012/13 season.
(DOCX)

S2 Table Aggregated measures including 2012/13 season. Mean (SD) in Squared Error, Absolute Proportional Error and Absolute Error for all locations. Unlike Table 1 and S1 Table, this includes 2012/13 season.
(DOCX)

S3 TableMAPE and MAE in one-week ahead estimates.*Change* column indicates percentage reduction in mean error by regressing GFT on lagged ILIp and lagged GFT. 2012/13 was excluded while aggregating overall and by region. Paired Wilcoxon signed rank tests for the hypothesis that errors in GFT (Z) are *greater* than errors in corrected GFT ($\hat{Z}$) were performed; cases where p > .05 are denoted by * and † for MAPE and MAE respectively.(DOCX)

S4 Table MAPE and MAE of near-term forecasts. The lower error in each row is underscored. An asterisk (*) indicates P > .05 with a paired Wilcoxon signed rank test for MAPE and † indicates P > .05 for MAE.
(DOCX)

S5 Table MSE, MAPE and MAE of near-term forecasts generated for *ILIp* and *ILIp+GFT*. The lower error in each row is underlined. Unlike Table 3 and S4 Table, this excludes 2012/13 season. Disaggregation by season is not shown as they are identical to errors reported in Table 3 and S4 Table.
(DOCX)

S6 Table MSE of near-term forecasts generated for *ILIp* and *ILIp+uncorrGFT*. The last two columns show mean squared errors with the 2012/13 season excluded from the aggregations. Due to the large errors in GFT during the 2012/13 season, in aggregations that include forecast errors from this season all other forecasts are overwhelmed and *ILIp* models almost always outperform. With 2012/13 excluded errors between *ILIp* and *ILIp+ uncorrGFT* are comparable.
(DOCX)

S1 Fig Absolute proportional errors from GFT (*x*-axis) and ILIp during the 2014/15 season. The green data point shows the error during week of maximum weekly ILIf—the peak week—and the remaining data points are color coded by their distance from peak week. The black triangle shows the mean error for the entire season. 2014/15 was the only season for which GFT estimates were generated with the final version of the GFT model.
(TIF)

S2 Fig Squared errors of GFT estimates and ILIp. *x*-axis shows week relative to peak, with the negative sign indicating weeks preceding peak. The box shows the interquartile range, the horizontal line indicates the median.
(TIF)

S3 Fig MAPE of GFT observed in US states. The top left panel, *Overall*, shows average errors across 5 seasons and each of the other panels is limited to one season. The data points are color coded by population size and ordered by *overall* error (high to low). The black line shows the errors from corresponding HHS regions.
(TIF)

S4 Fig Comparison of estimates and errors at US national during 5 seasons. A) Plot of estimates from ILIf (in black), ILIp (in blue), GFT (in red) and Corrected GFT (in orange). The vertical line indicates the week of peak ILIf; B) Corresponding errors relative to ILIf as reference.
(TIF)

S5 Fig MAPE of near term forecasts for *ILIp* and *ILIp+GFT* models. The data points are color coded by target. Points below the diagonal (broken black line) indicate instances where forecast quality improved with the use of GFT. Each panel is for one of the locations.
(TIF)

S1 Data An archive of near-term forecasts and corresponding errors *ILIp* and *ILIp+GFT* models. (RDATA)
