# Supplementary material for: Reappraising the utility of Google Flu Trends
Source: PLoS Comput Biol. 2019 Aug 2;15(8):e1007258. doi: 10.1371/journal.pcbi.1007258 (PMC6693776; doi:10.1371/journal.pcbi.1007258)
Supplement: S1 Table — Mean (standard deviation, [25th–75th percentile]) for the entire study period, disaggregated by location and season. US national has ILIp has 11 fewer dates than the regions. Overall and location aggregations exclude 2012/13 season. (DOCX) [file pcbi.1007258.s001.docx]

|  | **MAE** | | **MAPE** | |
| --- | --- | --- | --- | --- |
|  | **GFT** | **ILIp** | **GFT** | **ILIp** |
| Overall | 0.436 (0.42, [.14 - .59]) | 0.241 (0.29, [.06 - .3]) | 0.249 (0.21, [.09, .34]) | 0.136 (0.14, [.04 - .18]) |
| National* | 0.269 (0.26, [.07 - .41]) | 0.136 (0.11, [.05 - .19]) | 0.132 (0.12, [.04 - .17]) | 0.076 (0.06, [.02 - .12]) |
| Region 1 | 0.239 (0.21, [.07 - .38]) | 0.139 (0.14, [.04 - .2]) | 0.212 (0.15, [.08 - .29]) | 0.127 (0.11, [.04 - .18]) |
| Region 2 | 0.568 (0.44, [.24 - .82]) | 0.296 (0.31, [.09 - .37]) | 0.273 (0.19, [.13 - .37]) | 0.131 (0.11, [.05 - .18]) |
| Region 3 | 0.498 (0.31, [.27 - .68]) | 0.204 (0.16, [.09 - .28]) | 0.321 (0.23, [.14 - .48]) | 0.127 (0.1, [.05 - .19]) |
| Region 4 | 0.391 (0.36, [.15 - .46]) | 0.139 (0.13, [.05 - .18]) | 0.201 (0.13, [.09 - .3]) | 0.079 (0.07, [.03 - .1]) |
| Region 5 | 0.299 (0.24, [.1 - .43]) | 0.117 (0.1, [.04 - .16]) | 0.231 (0.2, [.06 - .33]) | 0.079 (0.06, [.03 - .12]) |
| Region 6 | 0.587 (0.61, [.18 - .82]) | 0.302 (0.3, [.09 - .41]) | 0.18 (0.15, [.07 - .26]) | 0.102 (0.11, [.03 - .13]) |
| Region 7 | 0.516 (0.58, [.12 - .64]) | 0.24 (0.23, [.09 - .3]) | 0.268 (0.17, [.11 - .39]) | 0.155 (0.12, [.08 - .21]) |
| Region 8 | 0.28 (0.24, [.11 - .38]) | 0.144 (0.15, [.02 - .26]) | 0.218 (0.12, [.12 - .31]) | 0.147 (0.18, [.02 - .26]) |
| Region 9 | 0.675 (0.49, [.26 - .95]) | 0.658 (0.5, [.22 - .96]) | 0.238 (0.15, [.11 - .35]) | 0.255 (0.19, [.07 - .4]) |
| Region 10 | 0.478 (0.33, [.23 - .63]) | 0.27 (0.31, [.05 - .38]) | 0.47 (0.39, [.19 - .64]) | 0.213 (0.23, [.04 - .31]) |
| 2009/10 | 0.401 (0.34, [.16 - .51]) | 0.303 (0.32,[.09 - .39]) | 0.271 (0.2, [.11 - .39]) | 0.204 (0.17, [.07 - .31]) |
| 2010/11 | 0.541 (0.5, [.17 - .75]) | 0.271 (0.33, [.06 - .34]) | 0.254 (0.17, [.12 - .37]) | 0.14 (0.14, [.04 - .2]) |
| 2011/12 | 0.488 (0.34, [.24 - .67]) | 0.242 (0.32, [.06-.27]) | 0.338 (0.24, [.17 - .47]) | 0.154 (0.17, [.05 - .18]) |
| 2012/13 | 1.496 (1.88, [.34 – 1.63]) | 0.244 (0.32, [.06 - .28]) | 0.622 (0.63, [.24 - .77]) | 0.113 (0.13, [.03 - .14]) |
| 2013/14 | 0.394 (0.37, [.1 - .58]) | 0.189 (0.22, [.05 - .22]) | 0.239 (0.27, [.06 - .31]) | 0.11 (0.12, [.03 - .14]) |
| 2014/15 | 0.354 (0.46, [.1 - .43]) | 0.221 (0.26, [.05 - .28]) | 0.155 (0.13, [.06 - .22]) | 0.094 (0.08, [.03 - .14]) |
