# Supplementary material for: Reappraising the utility of Google Flu Trends
Source: PLoS Comput Biol. 2019 Aug 2;15(8):e1007258. doi: 10.1371/journal.pcbi.1007258 (PMC6693776; doi:10.1371/journal.pcbi.1007258)
Supplement: S2 Table — Mean (SD) in Squared Error, Absolute Proportional Error and Absolute Error for all locations. Unlike Table 1 and S1 Table, this includes 2012/13 season. (DOCX) [file pcbi.1007258.s002.docx]

|  | **Squared Error**  **Mean (std.dev)** | | **Abs Prop Error**  **Mean (std.dev)** | | **Absolute Error**  **Mean (std. dev)** | |
| --- | --- | --- | --- | --- | --- | --- |
|  | **GFT** | **ILIp** | **GFT** | **ILIp** | **GFT** | **ILIp** |
| Overall | 1.295 (6.72) | 0.147 (0.43) | 0.313 (0.35) | 0.132 (0.14) | 0.619 (0.96) | 0.242 (0.3) |
| National* | 1.04 (4.48) | 0.033 (0.05) | 0.216 (0.24) | 0.078 (0.06) | 0.524 (0.88) | 0.145 (0.11) |
| Region 1 | 1.492 (11.18) | 0.042 (0.1) | 0.287 (0.39) | 0.122 (0.1) | 0.431 (1.15) | 0.14 (0.15) |
| Region 2 | 1.419 (6.06) | 0.174 (0.36) | 0.292 (0.24) | 0.132 (0.11) | 0.699 (0.97) | 0.298 (0.29) |
| Region 3 | 1.122 (3.66) | 0.076 (0.15) | 0.398 (0.31) | 0.128 (0.1) | 0.717 (0.78) | 0.217 (0.17) |
| Region 4 | 0.958 (3.6) | 0.035 (0.09) | 0.271 (0.25) | 0.072 (0.07) | 0.582 (0.79) | 0.131 (0.13) |
| Region 5 | 1.067 (5.13) | 0.024 (0.04) | 0.31 (0.3) | 0.077 (0.06) | 0.526 (0.89) | 0.118 (0.1) |
| Region 6 | 2.892 (12.03) | 0.157 (0.35) | 0.25 (0.24) | 0.093 (0.1) | 0.935 (1.42) | 0.279 (0.28) |
| Region 7 | 0.588 (1.36) | 0.105 (0.27) | 0.258 (0.16) | 0.151 (0.12) | 0.522 (0.56) | 0.238 (0.22) |
| Region 8 | 0.26 (1.14) | 0.037 (0.07) | 0.22 (0.14) | 0.129 (0.17) | 0.321 (0.4) | 0.13 (0.14) |
| Region 9 | 1.717 (7.66) | 0.768 (1.04) | 0.253 (0.23) | 0.272 (0.2) | 0.788 (1.05) | 0.706 (0.52) |
| Region 10 | 1.746 (7.34) | 0.155 (0.36) | 0.697 (0.69) | 0.196 (0.22) | 0.777 (1.07) | 0.252 (0.3) |
