# Supplementary material for: Reappraising the utility of Google Flu Trends
Source: PLoS Comput Biol. 2019 Aug 2;15(8):e1007258. doi: 10.1371/journal.pcbi.1007258 (PMC6693776; doi:10.1371/journal.pcbi.1007258)
Supplement: S3 Table — Change column indicates percentage reduction in mean error by regressing GFT on lagged ILIp and lagged GFT. 2012/13 was excluded while aggregating overall and by region. Paired Wilcoxon signed rank tests for the hypothesis that errors in GFT (Z) are greater than errors in corrected GFT (Z^) were performed; cases where p > .05 are denoted by * and † for MAPE and MAE respectively. (DOCX) [file pcbi.1007258.s003.docx]

|  | **MAPE** | | | | **MAE** | | | |
| --- | --- | --- | --- | --- | --- | --- | --- | --- |
|  | **ILIp** | **GFT**  **(Z)** | **Corrected GFT (**$\hat{\boldsymbol{Z}}$**)** | **Change,%** $\boldsymbol{(Z-}\hat{\boldsymbol{Z}}\boldsymbol{)/Z}$ | **ILIp** | **GFT**  **(Z)** | **Corrected GFT (**$\hat{\boldsymbol{Z}}$**)** | **Change,%** $\boldsymbol{(Z-}\hat{\boldsymbol{Z}}\boldsymbol{)/Z}$ |
| Overall | .179 | .247 | .170 | 31 | .354 | .445 | .314 | 29 |
| National | .115 | .140 | .086 | 39 | .252 | .285 | .195 | 32 |
| Region 1 | .165 | .219 | .160 | 27 | .192 | .264 | .181 | 31 |
| Region 2 | .161 | .298 | .181 | 39 | .381 | .634 | .380 | 40 |
| Region 3 | .169 | .333 | .175 | 47 | .336 | .522 | .340 | 35 |
| Region 4 | .143 | .199 | .131 | 34 | .315 | .397 | .275 | 31 |
| Region 5 | .138 | .207 | .132 | 36 | .258 | .288 | .222 | 23 |
| Region 6 | .122 | .188 | .119 | 37 | .492 | .634 | .435 | 31 |
| Region 7*† | .244 | .252 | .242 | 4 | .396 | .485 | .392 | 19 |
| Region 8 | .170 | .210 | .166 | 21 | .230 | .288 | .205 | 29 |
| Region 9 | .260 | .218 | .169 | 22 | .686 | .611 | .448 | 27 |
| Region 10 | .278 | .457 | .310 | 32 | .355 | .486 | .378 | 22 |
| 2010/11 | .197 | .257 | .163 | 37 | .394 | .542 | .321 | 41 |
| 2011/12 | .179 | .338 | .190 | 44 | .284 | .489 | .273 | 44 |
| 2012/13 | .166 | .614 | .234 | 62 | .402 | 1.480 | .530 | 64 |
| 2013/14 | .179 | .236 | .165 | 30 | .342 | .395 | .306 | 23 |
| 2014/15*† | .161 | .164 | .163 | 1 | .394 | .359 | .353 | 2 |
