# Supplementary material for: Reappraising the utility of Google Flu Trends
Source: PLoS Comput Biol. 2019 Aug 2;15(8):e1007258. doi: 10.1371/journal.pcbi.1007258 (PMC6693776; doi:10.1371/journal.pcbi.1007258)
Supplement: S4 Table — The lower error in each row is underscored. An asterisk (*) indicates P > .05 with a paired Wilcoxon signed rank test for MAPE and † indicates P > .05 for MAE. (DOCX) [file pcbi.1007258.s004.docx]

|  | **MAPE** | | **MAE** | |
| --- | --- | --- | --- | --- |
|  | **ILIp** | **ILIp + GFT** | **ILIp** | **ILIp + GFT** |
| Overall | 0.257 | 0.252 | 0.537 | 0.495 |
| 1 week ahead*^†^ | 0.176 | 0.183 | 0.363 | 0.357 |
| 2 week ahead | 0.227 | 0.217 | 0.487 | 0.441 |
| 3 week ahead | 0.282 | 0.272 | 0.599 | 0.541 |
| 4 week ahead | 0.341 | 0.334 | 0.700 | 0.643 |
| National | 0.164 | 0.152 | 0.416 | 0.384 |
| Region 1 | 0.221 | 0.212 | 0.305 | 0.294 |
| Region 2*^†^ | 0.209 | 0.215 | 0.527 | 0.501 |
| Region 3*^†^ | 0.237 | 0.242 | 0.544 | 0.539 |
| Region 4*^†^ | 0.257 | 0.262 | 0.590 | 0.544 |
| Region 5^†^ | 0.199 | 0.225 | 0.406 | 0.448 |
| Region 6 | 0.175 | 0.172 | 0.733 | 0.677 |
| Region 7 | 0.401 | 0.394 | 0.643 | 0.591 |
| Region 8 | 0.248 | 0.226 | 0.401 | 0.345 |
| Region 9 | 0.305 | 0.208 | 0.845 | 0.590 |
| Region 10 | 0.405 | 0.460 | 0.502 | 0.536 |
| 2010/11 | 0.292 | 0.248 | 0.587 | 0.496 |
| 2011/12* | 0.234 | 0.238 | 0.353 | 0.332 |
| 2012/13^†^ | 0.251 | 0.281 | 0.619 | 0.626 |
| 2013/14 | 0.252 | 0.241 | 0.507 | 0.468 |
| 2014/15 | 0.254 | 0.249 | 0.616 | 0.551 |
