# Supplementary material for: Reappraising the utility of Google Flu Trends
Source: PLoS Comput Biol. 2019 Aug 2;15(8):e1007258. doi: 10.1371/journal.pcbi.1007258 (PMC6693776; doi:10.1371/journal.pcbi.1007258)
Supplement: S5 Table — The lower error in each row is underlined. Unlike Table 3 and S4 Table, this excludes 2012/13 season. Disaggregation by season is not shown as they are identical to errors reported in Table 3 and S4 Table. (DOCX) [file pcbi.1007258.s005.docx]

|  | **MSE** | | **MAPE** | | **MAE** | |
| --- | --- | --- | --- | --- | --- | --- |
|  | **ILIp** | **ILIp + GFT** | **ILIp** | **ILIp + GFT** | **ILIp** | **ILIp + GFT** |
| Overall | 0.693 | 0.541 | 0.258 | 0.244 | 0.517 | 0.463 |
| 1 week ahead | 0.301 | 0.215 | 0.179 | 0.170 | 0.354 | 0.314 |
| 2 week ahead | 0.563 | 0.409 | 0.229 | 0.213 | 0.472 | 0.416 |
| 3 week ahead | 0.835 | 0.648 | 0.284 | 0.268 | 0.576 | 0.515 |
| 4 week ahead | 1.075 | 0.892 | 0.340 | 0.326 | 0.667 | 0.607 |
| National | 0.399 | 0.324 | 0.158 | 0.137 | 0.394 | 0.340 |
| Region 1 | 0.207 | 0.130 | 0.228 | 0.203 | 0.293 | 0.247 |
| Region 2 | 0.569 | 0.501 | 0.211 | 0.222 | 0.521 | 0.506 |
| Region 3 | 0.712 | 0.621 | 0.235 | 0.237 | 0.500 | 0.487 |
| Region 4 | 0.845 | 0.598 | 0.248 | 0.217 | 0.561 | 0.475 |
| Region 5 | 0.483 | 0.439 | 0.206 | 0.211 | 0.394 | 0.383 |
| Region 6 | 1.552 | 1.175 | 0.178 | 0.166 | 0.726 | 0.638 |
| Region 7 | 0.881 | 0.743 | 0.430 | 0.431 | 0.613 | 0.583 |
| Region 8 | 0.360 | 0.272 | 0.246 | 0.235 | 0.358 | 0.326 |
| Region 9 | 1.026 | 0.589 | 0.295 | 0.211 | 0.810 | 0.588 |
| Region 10 | 0.593 | 0.558 | 0.404 | 0.418 | 0.518 | 0.521 |
