# Supplementary material for: Reappraising the utility of Google Flu Trends
Source: PLoS Comput Biol. 2019 Aug 2;15(8):e1007258. doi: 10.1371/journal.pcbi.1007258 (PMC6693776; doi:10.1371/journal.pcbi.1007258)
Supplement: S6 Table — The last two columns show mean squared errors with the 2012/13 season excluded from the aggregations. Due to the large errors in GFT during the 2012/13 season, in aggregations that include forecast errors from this season all other forecasts are overwhelmed and ILIp models almost always outperform. With 2012/13 excluded errors between ILIp and ILIp+ uncorrGFT are comparable. (DOCX) [file pcbi.1007258.s006.docx]

|  | **ILIp** | **ILIp + uncorrGFT** | **ILIp** | **ILIp + uncorrGFT** |
| --- | --- | --- | --- | --- |
| Overall | 0.761 | 1.487 | 0.693 | 0.687 |
| 1 week ahead | 0.327 | 1.445 | 0.301 | 0.382 |
| 2 week ahead | 0.611 | 1.188 | 0.563 | 0.505 |
| 3 week ahead | 0.907 | 1.489 | 0.835 | 0.802 |
| 4 week ahead | 1.199 | 1.827 | 1.075 | 1.056 |
| National | 0.452 | 1.861 | 0.399 | 0.507 |
| Region 1 | 0.248 | 1.039 | 0.207 | 0.149 |
| Region 2 | 0.605 | 1.274 | 0.569 | 0.742 |
| Region 3 | 0.857 | 1.191 | 0.712 | 0.671 |
| Region 4 | 0.969 | 2.611 | 0.845 | 0.960 |
| Region 5 | 0.526 | 1.347 | 0.483 | 0.458 |
| Region 6 | 1.554 | 3.036 | 1.552 | 1.676 |
| Region 7 | 1.013 | 0.940 | 0.881 | 0.864 |
| Region 8 | 0.445 | 0.356 | 0.360 | 0.268 |
| Region 9 | 1.149 | 1.612 | 1.026 | 0.751 |
| Region 10 | 0.553 | 1.094 | 0.593 | 0.507 |
| 2010/11 | 0.747 | 0.725 |  |  |
| 2011/12 | 0.268 | 0.382 |  |  |
| 2012/13 | 1.035 | 4.740 |  |  |
| 2013/14 | 0.643 | 0.671 |  |  |
| 2014/15 | 1.091 | 0.952 |  |  |
