# Supplementary figures and images for: Reappraising the utility of Google Flu Trends
Source: PLoS Comput Biol. 2019 Aug 2;15(8):e1007258. doi: 10.1371/journal.pcbi.1007258 (PMC6693776; doi:10.1371/journal.pcbi.1007258)

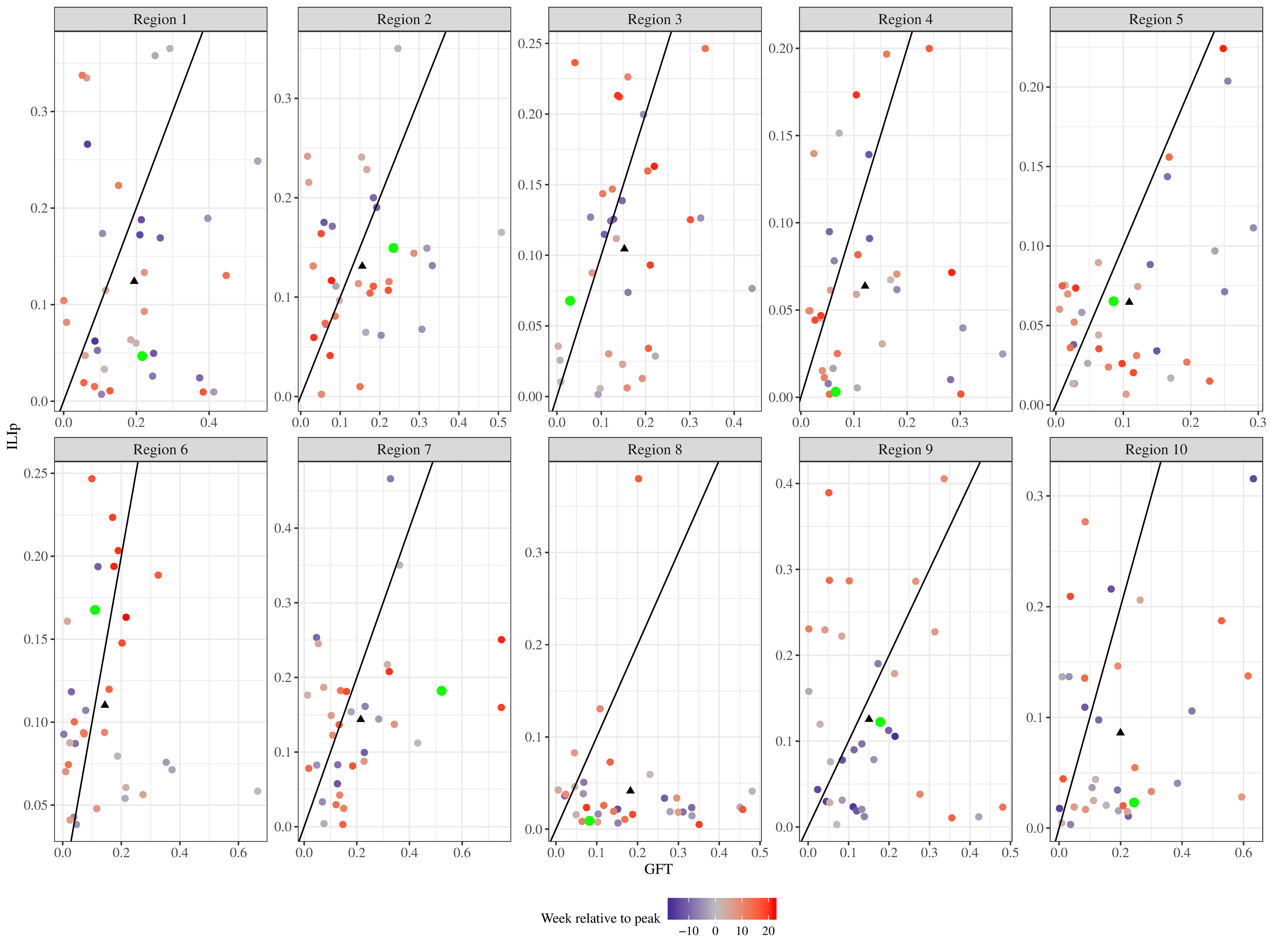

Supplement: S1 Fig — The green data point shows the error during week of maximum weekly ILIf—the peak week—and the remaining data points are color coded by their distance from peak week. The black triangle shows the mean error for the entire season. 2014/15 was the only season for which GFT estimates were generated with the final version of the GFT model. (TIF) [file pcbi.1007258.s007.tif]

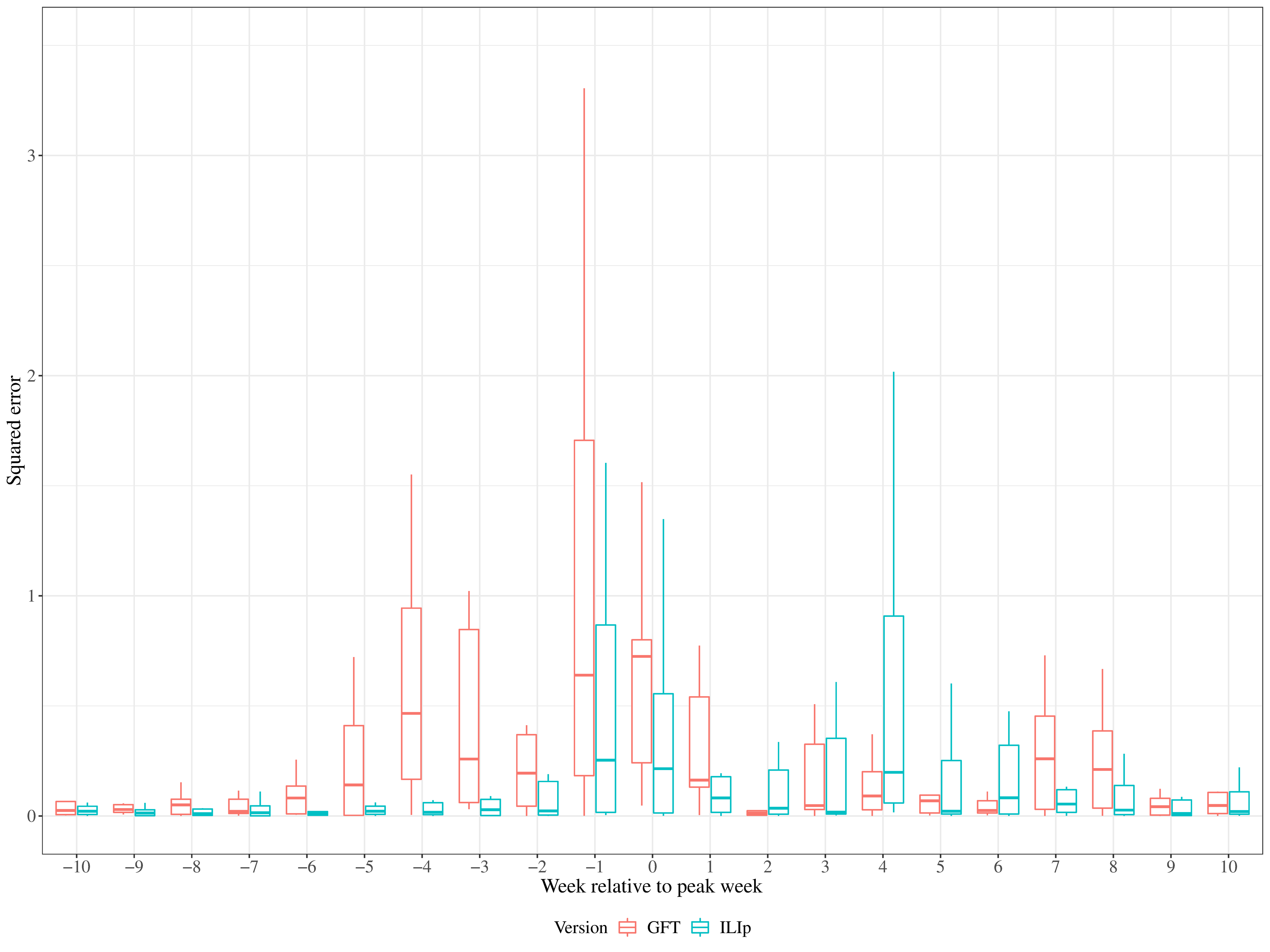

Supplement: S2 Fig — x-axis shows week relative to peak, with the negative sign indicating weeks preceding peak. The box shows the interquartile range, the horizontal line indicates the median. (TIF) [file pcbi.1007258.s008.tif]

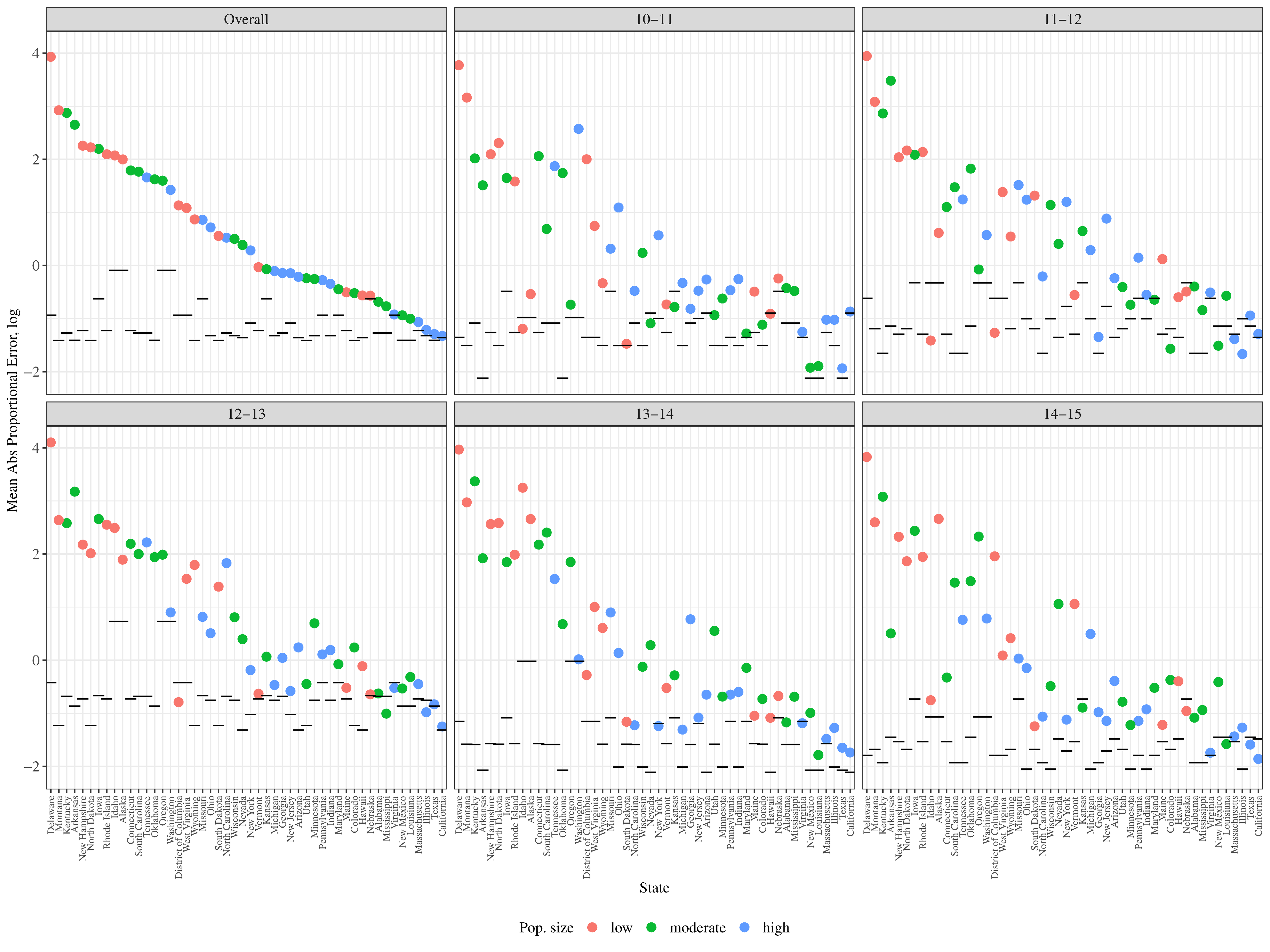

Supplement: S3 Fig — The top left panel, Overall, shows average errors across 5 seasons and each of the other panels is limited to one season. The data points are color coded by population size and ordered by overall error (high to low). The black line shows the errors from corresponding HHS regions. (TIF) [file pcbi.1007258.s009.tif]

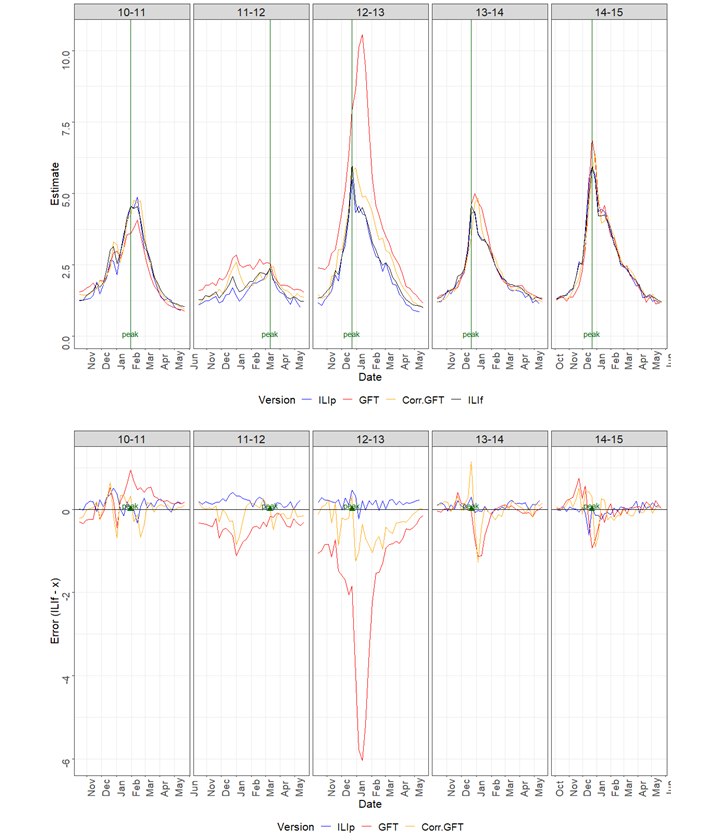

Supplement: S4 Fig — A) Plot of estimates from ILIf (in black), ILIp (in blue), GFT (in red) and Corrected GFT (in orange). The vertical line indicates the week of peak ILIf; B) Corresponding errors relative to ILIf as reference. (TIF) [file pcbi.1007258.s010.tif]

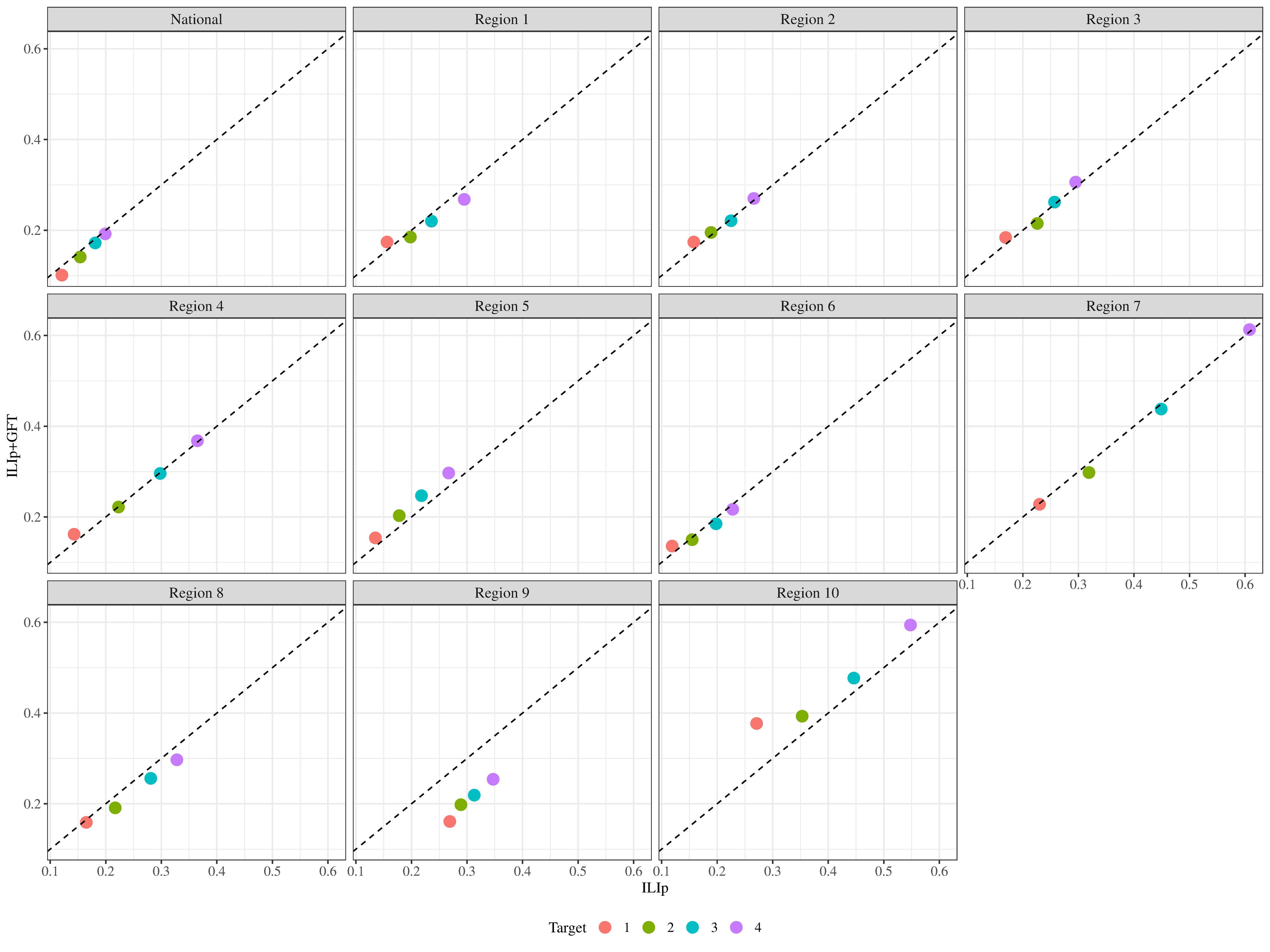

Supplement: S5 Fig — The data points are color coded by target. Points below the diagonal (broken black line) indicate instances where forecast quality improved with the use of GFT. Each panel is for one of the locations. (TIF) [file pcbi.1007258.s011.tif]
